# Oxidative Stress and Benefits of Antioxidant Agents in Acute and Chronic Hepatitis

**DOI:** 10.5812/hepatmon.837

**Published:** 2012-03-28

**Authors:** Mukaddes Esrefoglu

**Affiliations:** 1 Department of Histology and Embryology, Medical Faculty, Bezmialem Vakif University, Istanbul, Turkey

**Keywords:** Antioxidants, Oxidative Stress, Hepatitis

## Abstract

**Context:**

Oxidative damage due to oxidative stress is the failure of the cell's defense against the deleterious effects of harmful agents by means of its numerous autoprotective mechanisms. oxidative stress is a key impairment induced by various conditions, including atherosclerosis, hypertension, ischemia-reperfusion, hepatitis, pancreatitis, cancer, and neurodegenerative diseases.

**Evidence Acquisition:**

Oxidative stress is a common pathogenetic mechanism contributing to the initiation and progression of hepatic damage in cases of inflammatory liver disorders, including acute and chronic hepatitis. Antioxidant administration is a good therapeutic strategy for the treatment of hepatitis.

**Results:**

Our comprehensive review of the literature revealed that contradictory results have been obtained with many antioxidants and antioxidant agents.

**Conclusion:**

Since clinical studies to date have generally involved testing of the effects of antioxidant mixtures containing more than 2 antioxidants and also have been limited because of toxic effects of high doses of some antioxidants, antioxidant therapy for acute and chronic hepatitis needs further study.

## 1. Context

### 1.1. What is Oxidative Stress?

Oxidative damage due to oxidative stress is the failure of the cell's defense against the deleterious effects of harmful agents. The cell has numerous protective defense mechanisms against threatening factors that are induced by its adaptive mechanisms as well as by an increase in its synthetic and metabolic activities. For instance, if the target of the injury-causing agent is protein synthesis,which may result in a deficiency of structural or the other types of proteins (i.e. enzymes, pumps, transport proteins, and channels), the cell increases its synthetic activity if the protein synthesis apparatus is not damaged. If the target is energy metabolism, which may result in energy depletion, the cell increases the production of ATP via alternate pathways, such as anaerobic glycolysis, unless cellular glycogen content is depleted. These are compensatory responses of the cell to protect itself from damage and death. If the cell's defense fails, reversible damage transforms into irreversible damage,ultimately causing cell death. Most cells can tolerate a mild degree of oxidative stress because they have sufficient antioxidant defense capacity and repair systems that recognize and remove molecules damaged by oxidation. Cells generate energy by reducing molecular oxygen to water. During this process, small amounts of partially reduced reactive oxygen species (ROS) are produced as a byproduct of mitochondrial respiration. Some of these are free radicals that can damage many components of cells, including lipids, proteins, and nucleic acids [1][2]. Physiological levels of ROS are beneficial for cells. ROS can regulate transcription by activating the transcription of specific genes and can act in the immune system as effector molecules against pathogens. Many components of the cell, including mitochondria, endoplasmic reticulum, peroxisomes, membranes, and cytosol, can be sources of ROS. In general, there is a balance between the production of ROS and cellular antioxidant agents. The accumulation of low to moderate levels of ROS is generally counterbalanced by the cell's endogenous antioxidant defense system. Antioxidant agents act jointly to remove various ROS produced by free radical reactions. Indeed, antioxidant activity may be a consequence of ROS production. If the amount of ROS increases, and if these products destroy the apparatus by which antioxidant agents are produced, the cellular defense system is eventually incapacitated. It appears that higher levels of ROS induce necrotic cell death whereas lower levels lead to apoptosis.

### 1.2. How Can ROS Induce Hepatic Damage?

Oxidative stress is a key effect of various conditions, including anoxic/reoxygenation injury, autoimmune hepatitis, viral hepatitis, and alcoholic hepatitis [3]. Liver damage ranging from acute hepatitis to hepatocellular carcinoma is a result of apoptosis, necrosis, inflammation, immune response, fibrosis, ischemia, altered gene expression, and regeneration, all processes that involve hepatocytes and Kupffer, stellate, and endothelial cells [4][5]. Severe oxidative stress leads to hepatocyte necrosis, while less severe oxidative stress leads to apoptosis. ROS play an important role in the pathogenesis of inflammatory diseases, although the detailed mechanisms of ROS involvement are still controversial. Stimulation with cytokines leads to the generation of ROS by resident and infiltrating phagocytes and/or intracellularROS generation in all types of liver cells. In fact,ROS are essential for the host-defense functions of phagocytes and can modulate the formation of mediators involved in regulating sinusoidal blood flow and liver regeneration [6]. Intracellular parasitic viruses may remain clinically latent and without reactivation within host cells until the cellular redox balance is disturbed. Wide fluctuations in incubation periods for hepatitis B and C suggest that the viral activity can be determined by the redox state of cells. With more severe oxidative stress, the viral replication is more active with dispersion from lysed or apoptotic cells. Viruses use host cell synthetic processes for replication, leading to the disturbance of the normal physiological biochemistry of endoplasmic reticulum and mitochondria with augmented ROS generation and antioxidant depletion [5]. In children with chronic B or C hepatitis, low catalase (CAT) and superoxide dismutase (SOD) activity and evidence of increased lipid peroxidation indicate inadequate antioxidant defense [7]. Detection of an increase in malondialdehyde (MDA) levels, which is a product of lipid peroxidation in hepatitis B virus (HBV)-infected groups, indicates that oxidative stress is increased in HBV infection [8]. In acute and chronic active viral hepatitis, the markedly reduced plasma glutathione (GSH) levels increase on recovery [9]. HBV and hepatitis C virus (HCV) cause severe antioxidant depletion, and elevated levels of peroxidation products secondarily affect immune cells [5]. HCV infection is characterized by a systemic oxidative stress that is most likely caused by a combination of chronic inflammation, iron overload, liver damage, and proteins encoded by HCV. The increased generation of reactive oxygen and nitrogen species, together with the decreased antioxidant defense, promotes the development and progression of hepatic and extrahepatic complications of HCV infection Although both HCV and HBV cause hepatitis, HCV appears to be particularly potent at inducing oxidative stress, suggesting there are oxidative stress-inducing mechanisms that are unique to this virus [10]. Continued ethanol consumption increases the severity of liver damage, which progresses to frank inflammatory hepatitis and eventually to cirrhosis in humans and experimental baboons [11]. This progression is associated with oxidative stress, free radical generation, depletion of protective antioxidants (α-tocopherol, vitamin A, selenium, GSH, glutathione peroxidase [GSHPx]), reduced serum and liver zinc content, and hyperzincuria. Acute and chronic ethanol consumption increases ROS production, lowers cellular antioxidant levels, and enhances oxidative stress in many tissues, especially the liver [12]. Chronic ethanol consumption has long been known to repress mitochondrial functions [13]. The occurrence of DNA fragmentation in peripheral blood lymphocytes reflects a direct genotoxic effect of alcohol, HBV, and/or HCV and suggests that the same genotoxic effect may be induced in the liver and contribute to hepatocarcinogenesis [14].ROS, in particular superoxide anion, rapidly react with nitric oxide (NO) to form peroxynitrite, which may act as a dangerous molecule and induce lipid peroxidation. However, the formation of peroxynitrite may also be a protective pathway because through this mechanism NO acts as an ROS scavenger[4]. NO can also derive directly from cytokine-activated neutrophils and lymphocytes in the circulation. The liver contains an inducible form of NO-synthase (iNOS) in hepatocytes, cholangiocytes, Kupffer, and stellate cells, and the endothelial form (eNOS) in the endothelial cells [15]. In patients with chronic HCV infection, a direct correlation between iNOS induction and HCV-RNA titer has been documented. Therefore, in addition to oxidative stress,nitrosative stress may have a relevant role in the pathogenesis of chronic viral hepatitis [16]. NO plays a role in the pathophysiology of viral (chronic hepatitis B and C) and autoimmune (primary biliary cirrhosis and autoimmune hepatitis) liver diseases [17]. Mitochondria and cytochrome P450 enzymes are the main sources of ROS in hepatocytes acutely and/or chronically exposed to injury (via drugs, alcohol, viruses, etc.) [4]. Phagocytes such as macrophages and neutrophils are capable of destroying invading microorganisms and removing necrotic and cellular debris by producing ROS and releasing proteases. ROS released by neutrophils and other phagocytes can cause an intracellular oxidant stress in target cells, which can kill hepatocytes within 1-2 h in vivo without evidence of lipid peroxidation [18]. Recent experiments have shown that selective Kupffer cell-induced oxidant stress can cause hepatocellular injury in isolated perfused liver within 1-2 h [19]. Indeed, liver inflammation is initiated by Kupffer cells, and it involves the formation and release of many inflammatory mediators, which recruit neutropils,lymphocytes, and other inflammatory cells to damaged regions [20]. Hepatocytes and infiltrating inflammatory cells interact in a complex and multifactorial process through soluble mediators, surface receptors, and adhesion molecules [21]. In response to hepatocellular stress, pro-inflammatory cytokines, e.g., interleukin-1, interleukin-6, and tumor necrosis factor (TNF)-α are released. These agents stimulate neighboring hepatocytes and other nonparenchymal cells [22]. Pro-inflammatory cytokines such as TNF-α can induce the formation of ROS in hepatocytes [23], where they can play a role in a large variety of activities, including Ca2+ accumulation, circulatory and transport function, NO synthesis and metabolism, cytokine gene expression, caspase activity, growth factor synthesis and activity, DNA fragmentation, and Na+ influx. [4]. ROS can react with fatty acid chains of membrane phospholipids. Reaction products are mostly seen on the outer membrane of the mitochondria, suggesting that lipid peroxidation occurs mainly at the mitochondrial membrane and supporting the finding of remarkable mitochondrial changes in hepatocytes in alcoholic hepatitis, nonalcoholic steatohepatitis [24], and hepatitis C [25]. Some of the products of lipid peroxidation can lead to loss of integrity, resulting in necrosis. It is suggested that to selectively kill hepatocytes by lipid peroxidation, a combination of oxidant stress, iron mobilization, and depletion of cellular antioxidants is necessary [26]. Excessively high levels of iron are stored in the hepatocytes of patients with nonalcoholic steatohepatosis, alcoholic hepatitis, or hepatitis C. The over-accumulation of iron causes oxidative stress in the hepatocytes [27]. It is a fact that certain lipid peroxidation products are potent chemotactic factors for neutrophils and can modulate reactive oxygen formation [28]. Hepatocytes, with their more potent antioxidant systems, are less susceptible to oxidant stress than non-parenchymal cells [29]. Kupffer cells and endothelial cells are more exposed or sensitive to oxidative stress-related molecules [3]. Under inflammatory conditions, in which activated complement factors stimulate Kupffer cells to produce reactive oxygen in the hepatic vasculature, complement induces the enhanced sinusoidal release of GSH from hepatocytes [30]. Hepatocytes contain about 10% of the total body pool of GSH [31]. GSH in the space of Disse non-enzymatically reacts with hydrogen peroxide, peroxynitrite, and hypochlorous acid [32]. It also affects the transcription of proinflammatory/anti-inflammatory cytokine genes, liver regeneration through cytokine-mediated nuclear factor κ binding (NF-κB) induction, NO bioavailability, and energy metabolism [4]. Depletion of GSH may be both a reason for and a consequence of liver damage. GSH levels in the liver and circulation have been reported to be decreased in patients with alcoholic and viral cirrhosis and HCV-related chronic hepatitis [33][34]. Enhanced production of ROS and the altered GSH pool contribute to programmed death by activating gene expression for transcription factors such as NF-κB, leading to up-regulation of proinflammatory cytokines, chemokines, adhesion molecules, Fas ligands, survival genes, etc., which consecutively activate the cascade of caspases (apoptosis) or induce the release of cytochrome c and the depletion of ATP at the mitochondrial level (necrosis) [35][36]. Necrotic cell death is linked to the opening of the permeability transition pore and the onset of mitochondrial membrane permeability transition (MPT) [37], which leads to mitochondrial uncoupling and loss of membrane potential. A significant magnitude of oxidant stress causes oxidation of mitochondrial NAD(P)H and reactive oxygen formation by mitochondria, both of which increase mitochondrial free Ca2+ levels [38]. The MPT is induced by an increase of mitochondrial Ca2+ directly [39] or through the activation of mitochondrial serine proteases (calpains) [40]. In addition, cytosolic calpains promote membrane blebbing via degradation of cytoskeleton proteins [41]. The combination of these events leads to rapid necrotic cell death.The induction of the cytochrome P450 enzyme system, the endotoxin-induced cytokine expression in Kupffer cells, and neutrophil infiltration further enhance the production of ROS and deplete ATP reserves in hepatocytes. ROS generated from neutrophils migrate into the hepatocytes and potentiates the shift of apoptosis to necrosis [18]. Another contribution to cell death derives from the adhesion of cytotoxic lymphocytes that release proteases and perforin from cytotoxic granules, particularly in presence of ROS, which mediate the lymphocyte-Fas/Fas ligand interaction [42].

## 2. Evidence Acquisition

### 2.1. Are Antioxidants Effective in the Management of Acute and Chronic Hepatitis?

Since oxidative stress is a common pathogenetic mechanism contributing to initiation and progression of hepatic damage in inflammatory liver disorders, including acute and chronic hepatitis, antioxidants represent a good therapeutic strategy for the treatment of acute and chronic hepatitis. Many natural compounds have the ability to scavenge ROS, thereby reducing oxidative stress directly, or they may offer an indirect protection by activating endogenous defense systems [43]. Induction of important enzymes in a cell defense system, e.g., SOD, CAT,GSHPx, and others, seems conceivable because fully functional cells are more likely to respond than necrotic and damaged cells [44]. Significant reductions in serum levels of vitamins such as β-carotene, vitamin C, vitamin D, and vitamin E in patients with chronic viral hepatitis have been reported [45][46][47], but the underlying mechanisms have not been elucidated. Because GSH; ubiquinol; Zn; selenium; GSHPx; CAT; SOD; β carotene; and vitamins A, C, and E can be seriously depleted by the time viral hepatitis is diagnosed, their stores require urgent replenishment. Therefore, large doses of primary antioxidants should be the initial therapy to restore and thereafter to maintain serum and tissue concentrations at high normal values. Acute toxic and viral hepatitis are similar in histological features and in both situations the hepatic damage is due to oxidative stress, generation of free radicals, and lipid peroxidation. Thus management of viral hepatitis, as in the case of toxic hepatitis, necessitates early restoration of optimal concentrations of essential antioxidants in the liver and plasma. However, contradictory results have been obtained with some of the antioxidants; therefore, these antioxidant agents need to studied further. Moreover, the results of a meta-analysis in which beta carotene; vitamins A, C, and E; and selenium levels were assessed in 1225 participants of 20 randomized trials showed no evidence to support or refute the effectiveness of antioxidant supplements in patients with autoimmune liver diseases, viral hepatitis, alcoholic liver disease, and cirrhosis [48]. The results ofclinical studies about the efficiency of vitamins for acute and chronic viral hepatitis are generally controversial, and to determine whether a vitamin is beneficial is difficult. Although the antiviral activity of vitamins in chronic hepatitis B or C has notbeen demonstrated, some data indicate that vitamin E is able to reduce inflammation-induced oxidative stress and to restore immune response. To date, none of the vitamins havebeen recommended for the treatment of chronic viral hepatitis [49]. In fact, it is known that vitamin E enhances HCV RNA replication [50]. However, vitamin D2 inhibits HCV RNA replication [51]. Antioxidant supplements had no significant effect on end-of-treatment virological response in patients with chronic hepatitis B or chronic hepatitis C. Additionally, the antioxidant supplements had no significant effect on the sustained virological response in patients with chronic hepatitis C [48].

Vitamins C, A, and E are the most important antioxidants associated with cell-mediated immunity and toxic hepatitis [52]. Vitamin C protects cell components from free radical damage by reducing water soluble radicals, scavenging lipid-peroxidation-derived radicals, or reducing tocopherol radicals to tocopherol [53]. In human tissues, vitamin E is the principal lipid soluble chain-breaking antioxidant in mitochondria, microsomes, and lipoproteins [54]. Antioxidant therapy should include vitamins A, C, and E in combination (acting synergistically) at doses up to 6 times the recommended daily allowances, enhancing host immunity and resistance to pathogenic bacteria, viruses, and Chlamydia, partly by antioxidant effects and by facilitating optimal functioning of the host immune response [55][56].

## 3. Results

Selenium is more effective in preventing free radical production than vitamin E. Additionally vitamin C reconstitutes reduced vitamin E [57]. The important oxidation-reduction reactions of selenium compounds involve peroxide decomposition, free radical scavenging, and molecular repair of damaged sites [54]. Supplementation with a combination of selenium, ascorbic acid, beta-carotene, and alpha-tocopherol has been reported to be helpful ininhibiting the development of liver injury induced by D-galactosamine [58]. It has been shown that prophylactic administration of ubiquinone protects rat liver from toxic damage by D-galactosamine on both ultrastructural and cellular levels. Ubiquinone exerts an antioxidant effect, blocking the induction of lipid peroxidation both in intact and hepatic rats [41]. Beta-carotene, vitamin D2, and linoleic acid possessed anti-HCV activity in a cell culture system, and these nutrients were therefore considered potential candidates for enhancing the effects of interferon therapy [59]. Ebselen, an organoselenium compound and GSHPx analog, decreases oxidative stress and protects against stroke clinically. It also prevents early alcohol-induced liver injury, most likely by preventing oxidative stress, which decreases inflammation [60]. A large number of antioxidant interventions have been used against inflammatory liver injuries. Some of the antioxidant strategies have included treatment with important antioxidant enzymes in the cell defense system, including SOD; CAT; and GSH; vitamins A, C, and E; trace elements such as zinc and selenium; antioxidants isolated from plant and animal sources, including flavonoids (i.e. Silybummarianum, quercetin, kaempferol, and apigenin); phenolic compounds (i.e. canolol, green tea, rosemary, resveratrol), chemical antioxidants such as tirilazadmesylate; and ebselen, tannins, coumarins, xanthones, and alkaloids. Many natural compounds have the ability to scavenge ROS, thereby reducing oxidative stress directly, or they may offer indirect protection by activating endogenous defense systems [43]. Induction of important enzymes in the cell defense system, e.g.,SOD, CAT and GSHPx, is possible because fully functional cells are more likely to respond than necrotic and damaged cells [44]. Flavonoids are efficient suppressors of oxygen radicals because of their radical scavenging properties and are inducers of the cellular antioxidant system [29][43][61][62]. Vitamin E, vitamin C, ubiquinol, β-carotene, uric acid, thiols, and bilirubin also act as chain-breaking antioxidants [5]. Indeed, antioxidant drugs such as GSH, silymarin, vitamin E, and N-acetyl-cysteine can reduce oxidative and nitrosative stress, increase the GSH pool, and improve the activity of P450-enzymes, counteracting the progression of liver disease in some patients [63][64][65]. However, a randomized trial of antioxidant therapy, including N-acetylcysteine for 1 week, and vitamins A-E, biotin, selenium, zinc, manganese, copper, magnesium, folic acid, and coenzyme Q in acute alcoholic hepatitis did not improve 6-month survival in patients with severe alcoholic hepatitis [66]. Nonetheless, the plasma concentrations of vitamins A, D, E, folate (B12), thiamine (B1), and pyridoxine (B6) are low in patients with acute alcoholic hepatitis [67]. Recently, Phillips et al. [68] conducted a controlled trial in which the effects of prednisone were compared with those of an antioxidant cocktail (containing vitamins A, C, and E; selenium; allopurinol; desferrioxamine; and N-acetylcysteine) with intralipid as a membrane stabilizer. Antioxidants had an adverse effect on 30-day mortality. Mezey et al. [69] found no beneficial effect of vitamin E on liver function in patients with mild to moderate alcoholic hepatitis. Overall, the present data do not support antioxidants as a curative therapy for severe alcoholic hepatitis. However, Cox et al. [70] studied a combination of antioxidants (vitamins C and E) and ursodeoxycholic acid, a membrane-stabilizing agent, and found it as effective as corticosteroids.N-acetylcysteine can be safely administered to patients with fulminant liver failure of various causes and has been reported to reduce mortality in patients with alcoholic hepatitis [71]. De moine et al. [72] reported that N-acetylcysteine could be safely administered in cirrhosis patients with alcoholic hepatitis, with an improvement in some biological parameters (including significant decreases in aspartate transaminase, alkaline phosphatase, and prothrombin time). Zinc supplementation prevented hepatocyte apoptosis in mice subjected to long-term ethanol exposure, and the action of zinc is likely through suppression of oxidative stress and death receptor-mediated pathways [73]. Additionally, lecithin, vitamin-B complex, and tocopheryl acetate treatment have been reported to reduce oxidative stress by controlling ethanol-induced immunomodulatory activities and supporting antioxidant systems [74].Silymarin supplementation to antiviral therapy reduced oxidative stress in chronic hepatitis C patients [75]. A combination of 3 potent antioxidants (alpha-lipoic acid, silymarin, and selenium) induced marked improvement in clinical, laboratory, and histological test results of chronic HCV patients [76][77]. In a study involving 170 patients, 420 mg/day of silymarin administered for an average of 41 months resulted in a significant improvement in survival [78][79]. Silymarin inhibits HCV-induced oxidative stress by its antiviral action, reducing HCV replication, and by its antioxidative action, independently of suppression of virus. Because silymarin inhibits oxidative stress independently of suppression of virus activity, it is possible that the hepatoprotective actions of silymarin may extend to liver diseases of nonviral origin [80]. Silymarin has been reported to protect liver cells from a wide variety of toxins, including acetaminophen, ethanol, carbon tetrachloride, and D-galactosamine, as well as from ischemic injury, radiation, iron toxicity, and viral hepatitis [81][82]. A combination of antiviral and antioxidative therapies, including silymarin, ascorbic acid, lipoic acid, and alpha-tocopherol, may enhance the overall response rate in the patients with chronic hepatitis C [83]. Resveratrol, a dietary polyphenol, has been shown to possess potent antioxidant as well as anti-inflammatory properties and to mediate induction of antioxidant enzymes and modulate lipid metabolism while attenuating hepatic lipid peroxidation [84]. It increases hepatic GSH content, scavenges free radicals, and inhibits the transcription of NF-κB, which induces inflammation [85]. Resveratrol provides protection against liver damage produced by several hepatotoxins, including acetaminophen and ethanol [86][87]. However, resveratrol is not suitable as an antioxidant therapy for chronic hepatitis C because it significantly enhances HCV RNA replication [50]. Quercetin is a flavonoid antioxidant. Treatment of HCV infection with quercetin in tissue culture lowered intracellular viral accumulation and infectious particle production. Quercetin inhibits viral protein production independently of viral genome replication. Quercetin may allow for dissection of the viral life cycle and has potential therapeutic use to reduce virus production with low associated toxicity [88]. Additionally, dihydroquercetin has been shown to be beneficial as a hepatoprotective substance in the treatment of toxic hepatitis and liver fibrosis by enhancing antioxidant enzyme activity and decreasing the pro-oxidant effect [89][90]. Recently a mitochondria-targeted antioxidant-mitoquinone-has been reported to be effective indecreasing liver damage in patients with chronic hepatitis C [91]. This was the first report of a potential clinical benefit from the use of mitochondria-targeted antioxidants in humans. Ideally, a mitochondria-targeted antioxidant will be a pharmaceutically tractable, stable, small molecule with acceptable oral bioavailability that is selectively taken up by mitochondria within organs, where it is able to control oxidative damage, and in the ideal situation, can be recycled to the active antioxidant form [92].

## 4. Conclusions

Since contradictory results regarding many antioxidants exist, and clinical studies, to date, have generally involvedtesting the effects of antioxidant combinations containing more than 2 antioxidants and have been limited because of toxic effects of high doses of some antioxidants, antioxidant therapy in individuals with acute and chronic hepatitis needs further evaluation.
